# Violence Associated with Somatic Delusions

**DOI:** 10.7759/cureus.3186

**Published:** 2018-08-22

**Authors:** Sanya A Virani, John Sobotka, Navjot Brainch, Lama Bazzi

**Affiliations:** 1 Psychiatry, Maimonides Medical Center, Brooklyn, USA; 2 Psychiatry, Maimonides Medical Center, New York, USA

**Keywords:** somatic delusions, delusional disorder, violence risk assessment, premeditated violence

## Abstract

The Diagnostic and Statistical Manual of Mental Disorders, Fifth Edition (DSM-5) characterizes delusional disorder (DD) by the presence of delusions for longer than one month, without bizarre behavior or functional impairment. According to Kaplan and Saddock, the lifetime prevalence of DD (all subtypes) is about 0.2%. The persecutory subtype of delusional disorder (DD-PS) is the most common and the somatic subtype (DD-SS) is exceedingly rare.

We aim to describe two cases of patients with somatic delusions, both presenting as imminently dangerous and threatening. We also discuss one case that resulted from our extensive literature review where somatic delusions were implicated in elevating a mass shooter’s violence risk.

Both patients whose cases are presented were involuntarily hospitalized after their doctors called 911 to report that they were being threatened by a weapon. These patients had no established psychiatric diagnoses and were evaluated thoroughly and diagnosed with DD-SS. Both perceived that their physicians were indifferent to their needs and cited their frustration as the trigger for planning attacks on the doctors.

Unlike PS, SS is not traditionally described as increasing danger or risk of violence, and thorough risk assessments are not usually performed in DD-SS. We demonstrate that formal psychiatric violence risk assessments remain a useful tool to methodically stratify and effectively address risk, even in patients we do not typically expect to demonstrate premeditated violence.

## Introduction

False, fixed beliefs that are not in keeping with the culture are referred to as delusions [1]. The Diagnostic and Statistical Manual of Mental Disorders, Fifth Edition (DSM-5) characterizes delusional disorder (DD) by the presence of delusions for longer than one month, without bizarre behavior or functional impairment and not attributable to other mental illnesses like schizophrenia, schizophreniform disorder, or mood disorders [2].

Although its etiology is unclear, DD is seen in a number of non-psychiatric medical conditions, including those with neurological lesions of the basal ganglia and the temporal lobe [1-3]. Limited evidence does suggest that hyperdopaminergic states, similar to those in schizophrenia, have been implicated in DD. It has been hypothesized that a hyperdopaminergic state is strongly associated with paranoid symptoms and the persecutory subtype of delusional disorder (DD-PS) especially comprises the “dopamine psychosis,” which forms the basis for the state [4].

The somatic subtype has also been referred to as monosymptomatic hypochondriacal psychosis. The most common themes of somatic delusions are those related to infestation (including parasitosis); distortion of shape or displacement, absence, or malfunction of a body part; body pain and weakness; or loss of identity in which a body part may seem separate or alien.

The association of dangerousness with DD is not based solely on the tendencies to become violent but also suicidal. The DELIREMP study (the empirical redefinition of delusional disorder and its phenomenology) reported that suicidal thoughts and suicide attempts are more frequently found in the persecutory and somatic subtypes of DD [5]. The risk of suicide in the persecutory and somatic subtypes ranges from 8% to 21% [6]. The erotomanic, persecutory, and jealous subtypes were more associated with violence and though the basis for this is not defined in neurobiology, it is perhaps the nature of the delusion that makes people harboring them more prone to react violently [7].

To our knowledge, there is only one reported case of violence in a patient suffering from the somatic subtype of delusional disorder (DD-SS) [8]. In addition to reviewing this case, we will present two cases of patients with isolated somatic delusions who made threats of violence to their healthcare providers and were brought to the emergency room (ER) of a large community hospital in New York. We emphasize the importance of performing a thorough and comprehensive violence risk assessment in patients with DD, regardless of the subtype.

Cases of somatic delusions and mass shooting

A thorough search of the literature yielded one case by Sarteschi, published in 2016 [8], which described the sequence of events leading up to an attempted mass murder at a large psychiatric facility in 2012. The perpetrator suffered from DD-SS.

He was an intelligent and well-educated man with several degrees who premeditated an attempted mass shooting by buying guns well in advance and making detailed plans to attack the hospital he ended up targeting. He managed to injure several people and killed one before being shot by the police. The investigation into the crime revealed that he had exhibited a pattern of bizarre and aggressive behavior at home, school, and work and during interactions with family and medical professionals. He had once threatened a medical provider with a baseball bat, which led to the doctor dismissing him from his practice. The day before the shooting, he presented to an ER requesting pain medication and left without incident when he was told they could not treat him because he refused to answer their questions. Although violence is not usually associated with somatic delusions, this case demonstrates the magnitude of harm that can ensue, even in rare cases.

## Case presentation

Case 1: Ms. K, a 70-year-old woman who immigrated to the US at the age of 53, began complaining of watery eyes, chest pain, lower back and joint pain, leg cramps, and weakness. She harbored delusions of being afflicted with high blood pressure, uterine cancer, blood cancer with bone metastasis, brain cancer with extensive metastasis, and believed that her brain was "shrinking."

She first visited a cardiologist in 2013, complaining of intermittent episodes of chest pain over six months. An electrocardiogram (EKG) at the time showed bradycardia, a first-degree atrioventricular (AV) block and a left bundle branch block. At her sixth visit with the cardiologist, she mentioned non-specific somatic complaints, which she said were because of a "hematological problem." Five months later, she was evaluated for “renal hypertension” and imaging studies showed a renal cyst. While she did not follow up with the nephrologist, she continued to make hospital visits for persistent chest pain. A full medical workup was completed and found to be normal at every ER visit. Medical records from a prior ER visit revealed that she had made claims that the Russian military entered her residence and stole her urine, resulting in the disappearance of her kidneys.

Ms. K was brought to the ER by the police after she showed up with a can of gasoline and matches at her primary doctor's office and threatened to burn it down. She was irate and claimed that all of her doctors, in the US and in her home country, were concealing the fact that she had oncological issues. She vehemently denied any psychiatric illness, stating that these diagnoses appeared on her records as a result of a rumor started by an envious former colleague. She explained that because she had been a former practicing neurologist in her home country, she was confident that she had cancer. Upon repeated questioning, she admitted that in a final bid to receive the medical attention that she was rightfully due, she had devised the plan to burn down the doctor’s office.

While in the psychiatric inpatient unit, she remained somatically preoccupied and reported abdominal pain, lower back pain, and weakness, which she attributed to the metastatic spread of uterine cancer to her spine. Radiological imaging confirmed no evidence of uterine cancer, though a thickened endometrium was reported with recommendations for further testing by tissue sampling. Because Ms. K’s ability to make rational and reasonable decisions about her psychiatric and medical treatment was compromised by her delusions, the team sought and was granted a court order allowing them to treat her over her objection.

Case 2: Ms. M was a 64-year-old woman whose medical history was significant for several visits to different doctors seeking treatment for somatic complaints, including progressive blindness, headaches, hypertension, pelvic pain, vaginal swelling, lower extremity pain, and swelling. She was brought into the ER from her primary care doctor’s office for complaints of pelvic and leg swelling, which she attributed to poor diet. She expressed great concern over her problems being persistently ignored at all ERs she had been to.

Ms. M was found hiding in the closet of a primary care doctor’s office early in the morning before it was open for business. Apparently, she saw the janitor approaching and got scared. When the police were able to get into the closet, they discovered a knife, a pair of scissors, and a child's toy phone (which she had been using to call her doctor) in her possession. She revealed that she had gone to the office out of exasperation because she believed that no doctors were listening to her concerns.

She informed the team of the inpatient psychiatric unit about her history of hypertension and migraine headaches. All radiological imaging was negative for structural problems. She became increasingly irritable and accused the treatment team of not having her best interests in mind. She refused meals because she was convinced that they were poisoned and laced with psychiatric medication. Her persistent complaint of pelvic swelling and subsequent physical examination prompted a gynecology consult, which revealed a vaginal prolapse. She became fixated on the problem and demanded an immediate resolution of the issue. She continually refused psychiatric medications, interviews, blood pressure checks, and blood draws. The treatment team sought a court order and the judge ruled in favor of administering medications against her will, and she conceded and started taking them. In the days ensuing, her insight gradually improved, she became much calmer and less argumentative, and even accepted increased dosages, affirming that she understood the indication. While her somatic complaints did not dissipate, she improved to a point where she was deemed to no longer be a danger to herself or others and she was discharged.

## Discussion

Both patients described did not meet criteria for schizophrenia or borderline personality disorder. Hallucinations or negative symptoms, often the hallmarks of schizophrenia, were absent. Their delusions clearly revolved around bodily functions and sensations, and their speech and behavior were organized as evidenced by their ability to advocate for themselves to multiple providers. Also, no history of interpersonal conflicts or gross impulsivity was noted from the collateral information provided by close family members. Further, they did not struggle with issues of abandonment, self-mutilation, suicidal behaviors, or affective instabilities, and they consistently expressed only dissatisfaction with inadequate care.

The theme common to all three cases was escalating frustration with health care providers who all patients perceived as indifferent to their medical needs. Strongly held delusional beliefs, low frustration tolerance, and feelings of helplessness can collectively trigger patients to threaten or potentially harm health care providers. Herein lies the importance of performing thorough violence risk assessments in all psychiatric patients, even if their diagnosis is not traditionally associated with an increased risk of violence.

Psychotherapy, especially cognitive-behavioral therapy, could decrease anxiety and temporarily reduce a patient's need to escalate delusions or act on them, although these methods do not effectively ameliorate the delusions themselves. The issue also lies in the lack of experience with correctly identifying the rapid worsening of delusions with the potential for invoking impulsive behavior.

## Conclusions

Violence risk assessments are a helpful tool in estimating a patient’s risk of harming themselves or others. They are not, however, infallible. While several risk assessment and mitigation strategies to manage aggression in patients with schizophrenia have been identified, no tool has been specifically designed to evaluate patients with DD.

Also, to improve the accuracy of these assessments and communication and collaboration between healthcare providers, case managers, community outreach teams, and family members are key. Ensuring that the information obtained is comprehensively included in discharge summaries and shared with providers seeing the patients in different settings ensures that informed decision-making takes place. This can prevent the “silo effect” described by Sarteschi et al., which occurs when various healthcare providers, across multiple offices, have knowledge of worrisome behavior patterns, but no one single entity assesses them thoroughly for risk or has access to all past records.

Because patients with somatic delusions genuinely believe that their problems are physical, they are more likely to have multiple encounters with primary care doctors first, who would benefit from wider knowledge and training to manage these kinds of mental health issues and build therapeutic alliances to ensure regular follow-up and thereby mitigate violence risk.
